# Syringeable Self-Organizing Gels that Trigger Gold Nanoparticle Formation for Localized Thermal Ablation

**DOI:** 10.3390/pharmaceutics11020052

**Published:** 2019-01-26

**Authors:** Sonia Cabana-Montenegro, Silvia Barbosa, Pablo Taboada, Angel Concheiro, Carmen Alvarez-Lorenzo

**Affiliations:** 1 Departamento de Farmacología, Farmacia y Tecnología Farmacéutica, R+D Pharma Group (GI-1645), Facultad de Farmacia and Health Research Institute of Santiago de Compostela (IDIS), Universidade de Santiago de Compostela, 15782 Santiago de Compostela, Spain; scabmon@gmail.com (S.C.-M.); angel.concheiro@usc.es (A.C.); 2 Área de Física de la Materia Condensada, Facultad de Física, Universidade de Santiago de Compostela, 15782 Santiago de Compostela, Spain; silvia.barbosa@usc.es (S.B.); pablo.taboada@usc.es (P.T.)

**Keywords:** in situ gelling systems, photo-thermal therapy, gold reduction, localized heating effect, irradiation cycles, syringeable implant

## Abstract

Block copolymer dispersions that form gels at body temperature and that additionally are able to reduce a gold salt to nanoparticles (AuNPs) directly in the final formulation under mild conditions were designed as hybrid depots for photothermal therapy. The in situ gelling systems may retain AuNPs in the application zone for a long time so that localized elevations of temperature can be achieved each time the zone is irradiated. To carry out the work, dispersions were prepared covering a wide range of poloxamine Tetronic 1307:gold salt molar ratios in NaCl media (also varying from pure water to hypertonic solution). Even at copolymer concentrations well above the critical micelle concentration, the reducing power of the copolymer was maintained, and AuNPs were formed in few hours without extra additives. Varying the copolymer and NaCl concentrations allowed a fine tuning of nanoparticles’ shape from spherical to triangular nanoplates, which determined that the surface plasmon resonance showed a maximum intensity at 540 nm or at 1000 nm, respectively. The information gathered on the effects of (i) the poloxamine concentration on AuNPs’ size and shape under isotonic conditions, (ii) the AuNPs on the temperature-induced gelling transition, and (iii) the gel properties on the photothermal responsiveness of the AuNPs during successive irradiation cycles may help the rational design of one-pot gels with built-in temperature and light responsiveness.

## 1. Introduction

Gold nanoparticles (AuNPs) are receiving a great deal of attention because of their capability to perform as theranostic agents [1,2,3,4,5]. Their localized surface plasmon resonance (LSPR) allows for efficient conversion of radiation energy into heat, increasing the temperature of the surroundings remarkably but reversibly. Tuning the shape of AuNPs’ selective light absorption in the wavelength range of the first biological window is possible [6]. Moreover, AuNPs can be easily decorated with therapeutic molecules, shielding coatings, and specific cell ligands to combine passive and active targeting [7]. All of these properties are particularly suitable to provide chemotherapy and phototherapy (including thermal ablation) synergisms in the eradication of tumor cells and pathogen microorganisms [8,9,10,11]. 

Most research in the field has been focused on the intravenous administration of AuNPs for cancer therapy. However, relatively rapid clearance of AuNPs from the bloodstream and accumulation in the liver and spleen hampers the distribution towards the target site and obligates the administration of repeated injections [12,13]. Intratumoral injection of AuNPs has been also proposed as a way to solve some physiological barriers and concentrate the nanoparticles in the affected tissue [14]. In this case, precise positioning of the AuNPs nearby the affected cells is difficult, which, in turn, hinders the attainment of predictable/reproducible levels of attached therapeutic agents and temperature. To overcome this problem, implantable solid depots with dimensions similar to those of brachytherapy seeds were designed recently [15]. In that case, poly(ethylene oxide) (PEO)-coated spherical AuNPs (ranging from 5 to 50 nm) were encapsulated in calcium alginate rods (0.8 mm diameter × 4 mm length) to be administered using a pre-loaded needle approach. The calcium alginate rods provided sustained release of the AuNPs in the tumor tissue. Also, lately, it has been demonstrated that integration of AuNPs in polymer systems that cause the AuNPs to assembly in vivo forming large supramolecular structures allows the LSPR peaks to be tuned towards the near-infrared (NIR) window in biological tissue (650–1350 nm) for more efficient cancer photothermal therapy and imaging [16,17].

The aim of the present work was to design in situ gelling systems from block copolymers that can transform a gold salt into AuNPs directly in the final injectable formulation and then self-assemble under physiological conditions to render a gel depot that allows repeated photothermal therapy cycles in the injection site. The overall purpose is to simplify the development (in one step under mild conditions) of gel depots containing AuNPs that can fully develop their photothermal properties without loss of efficiency after several light irradiations. Relevantly, since there is no covalent grafting of the composite components, gel erosion in vivo after treatment would cause the dissociation of the assemblies and thus the release of single AuNPs avoiding compromise their clearance from the body [16,17]. Poly(ethylene oxide)-poly(propylene oxide)-poly(ethylene oxide), PEO–PPO–PEO, block copolymers (namely poloxamers) have been shown able to trigger gold reduction under certain conditions without the need for strong reductant agents and providing a stabilizing shell [18,19]. The size of the obtained AuNPs can be tuned by changing the molecular weight and concentration of the poloxamer, preferably below the critical micelle concentration (CMC). The reducing power of PEO blocks causes nuclei formation to occur quite rapidly, while the stabilizing contribution of PPO blocks adsorbed on the nuclei makes the subsequent growth more slowly [20]. Compared to the linear poloxamer copolymers, the X-shaped poloxamines formed by four arms of PEO–PPO blocks linked by an ethylenediamine group may offer additional features [21,22,23]. The presence of the tertiary amines cause the copolymer to exhibit both temperature and pH responsiveness [24,25,26,27]. Moreover, the amine groups may contribute to further binding and reduction of gold ions, while the looser self-assembly of the copolymer may expedite the growth of the nanoparticles. These features may allow for more precise regulation of the kinetic and thermodynamic parameters involved in AuNP formation, which, in turn, should facilitate fine tuning of the shape of the nanoparticles [21]. So far, most studies have been carried out with low molecular weight PEO–PPO–PEO copolymers at concentrations below or close to those typical of micelle formation, where the predominant species are unimers or single micelles. Differently, the purpose of our work was to use a biocompatible poloxamine of relatively high molecular weight (Tetronic 1307, T1307) covering concentrations up to those suitable for forming aqueous micellar dispersions that, once injected, can undergo the sol-to-gel transition at the physiological temperature. T1307 is formed by four arms of PEO–PPO blocks (72 EO units and 23 PO units, each arm) connected to a central ethylenediamine group [24]. A gold salt was added to the poloxamine dispersions at two different concentrations, and the spontaneous formation of nanoparticles without the addition of other reagents was monitored. Since syringeable formulations should be isotonic, the effects of the ionic strength on the size and shape of the AuNPs formed were also investigated. Compared to solid depots, in situ gelling systems can perfectly fill gaps in tumor resection areas (covering tumor borders) or form soft depots in tumor or infected tissues. Thus, it can be hypothesized that the obtained in situ formed gold-reductant gels may retain the AuNPs in the application site for a prolonged amount of time for full exploitation of their light-induced heating. Rational design requires an insight to be gained into the effects of (i) the poloxamine concentration on AuNPs’ size and shape, (ii) AuNPs on the temperature-induced gelling transition, and (iii) the gel properties on the responsiveness of the AuNPs. All these issues were investigated herein in detail.

## 2. Materials and Methods 

### 2.1. Materials

Tetronic 1307 (T1307; molecular weight 18,000; PEO content 70 wt%) was supplied by BASF Corporation (Mount Olive, NJ, USA) and used as received. Hydrogen tetrachloroaurate(III) (HAuCl_4_) and sodium chloride (NaCl) were from Sigma Aldrich (St Louis, MO, USA) and used without further purification. Distilled water was used for all experiments. All other reagents were analytical grade.

### 2.2. Gel Preparation and Nanoparticles Formation

T1307 solutions (0.25, 1, 5, 10, 15, 20, and 30 mM) were prepared in water and in 0.154 or 1.0 M NaCl aqueous medium by mixing under magnetic stirring in an ice-water bath. Aliquots of each T1307 solution (2 mL) in every medium were placed in glass vials. Then, HAuCl_4_ aqueous solution (0.2 mL; 0.5 or 5 mM) was added to the vials and mixed with vortex for 2 min. After that, solutions were kept in the fridge for 4 hours. The final HAuCl_4_ concentration was 0.05 or 0.5 mM. The systems were designed as T1307(*x*)/Au(*y*), where *x* and *y* represent the concentration (mM) of each component. Before measurements, all T1307/Au dispersions were stored for 24 h at 20 °C. The pH was recorded for all dispersions.

### 2.3. Nanoparticle Characterization

The UV-Vis spectra of T1307/Au dispersions were recorded in the 400 to 1100 nm range (Agilent 8453, Frankfurt am Main, Germany). The morphology of the nanoparticles was visualized in a scanning transmission electron microscope (STEM). Drops (5 µL) of T1307 dispersions with AuNPs were placed on grids covered with Fomvar film. After 30 s, the excess was carefully removed with a tip of filter paper. The samples were observed both with and without staining (with phosphotungstic acid) using a ZEISS UltraPlus FESEM apparatus fitted with a STEM detector (Oberkochen, Germany). Hydrodynamic particle size and zeta potential were measured in triplicate at room temperature using a Malvern Nano ZS (Worcestershire, UK) instrument. Before measurements, all solutions were sonicated for three minutes at room temperature to break up weak agglomerates. Thermal gravimetric analysis (TGA) was performed using a Discovery TGA 55 (TA Instruments, New Castle, DE, USA) and the sample dispersion was heated up to 450 °C at 10 °C/min and then was kept isothermal at 450 °C for 1 min.

### 2.4. Rheology

The storage or elastic (G’) and the loss or viscous (G’’) moduli of T1307 (10, 15, and 20 mM)/Au (0.05 or 0.5 mM) dispersions in water and in 0.154 and 1 M NaCl aqueous medium were evaluated, at least in duplicate, using a Rheolyst AR-1000N rheometer equipped with an AR2500 data analyzer, a Peltier plate, and a cone with 6 cm diameter (TA Instruments, Hertfordshire, UK). To determine the influence of temperature on both moduli, tests were carried out at 5 rad/s from 15 to 45 °C with a heating rate of 2 °C/min. 

### 2.5. Photothermal Measurements

Aliquots (2.2 mL) of T1307 (10, 15, and 20 mM)/Au (0.05 or 0.5 mM) dispersions were placed in 24-well plates and kept at 37 °C (using a temperature-controlled plate). The wells were irradiated with a 980 nm laser (Apollo Instruments, Inc. S10-976-1, Irvine, CA, USA) with a spot size of 1 cm^2^ and power of 0.5, 1, and 2 W/cm^2^ for 20 min at a distance of 8 cm from the laser. Temperature elevation was registered in the dispersion with a thermocouple (XS Instruments 3JKT, Carpi MO, Italy). Two independent experiments were carried out for each composition. Additionally, T1307 (15 and 20 mM)/Au (0.05 or 0.5 mM) dispersions were subjected to three successive irradiation cycles (8–12 min) at power densities of 0.5 and 2 W/cm^2^, and the temperature was recorded during the whole process.

## 3. Results and Discussion

### 3.1. Reduction of AuCl_4_^−^ Ions and Formation of Gold Nanoparticles

A first aim of this work was to elucidate whether the high molecular weight poloxamine T1307 is able to induce the reduction of gold ions under mild conditions, even when the copolymer concentration is well above the CMC and in the absence of any other chemicals, in order to preserve the previously demonstrated high biocompatibility of this block copolymer dispersions [25,28,29]. In the case of poloxamers, it has been shown that both the size and shape of metal nanoparticles are determined by competition between nucleation (metal ion reduction in bulk) and growth (metal ion reduction on nuclei) processes, which are controlled by the amphiphilic character of the block copolymer [19]. PEO blocks form crown ether-like domains that bind and reduce AuCl_4_^−^ ions, which triggers the formation of AuNPs. PPO blocks mediate copolymer adsorption onto the nanoparticles’ surfaces. This results in competition between AuCl_4_^−^ ion reduction in the bulk solution and on the particle surface, which, in turn, leads to an enhancement of the number of particles or an increase in particle size, respectively [18]. 

The formation of AuNPs in the presence of T1307 in media of different NaCl concentrations was monitored by recording the UV-visible spectrum. The gold salt was added at two different concentrations (0.05 and 0.5 mM final concentration) to T1307 (0.25, 1, 5, 10, 15, 20, and 30 mM) dispersions prepared in water and in 0.154 and 1.0 M NaCl aqueous mediums. The NaCl concentration was chosen to be the isotonic value (0.154 M) or a hypertonic value (1.0 M) that is still tolerable if small volumes are injected [30]. We have previously shown that increasing the NaCl concentration from 0 to 1 M notably decreases the CMC of T1307 (from 1% to 0.1%, i.e., from 0.55 to 0.05 mM); namely, the salt facilitates the copolymer self-assembly and also diminishes the gelling temperature [31]. Therefore, the use of NaCl may also help to elucidate whether the reducing power of the copolymer after self-assembly in micelles is similar to that of the unimers free in the medium.

**Figure 1:**
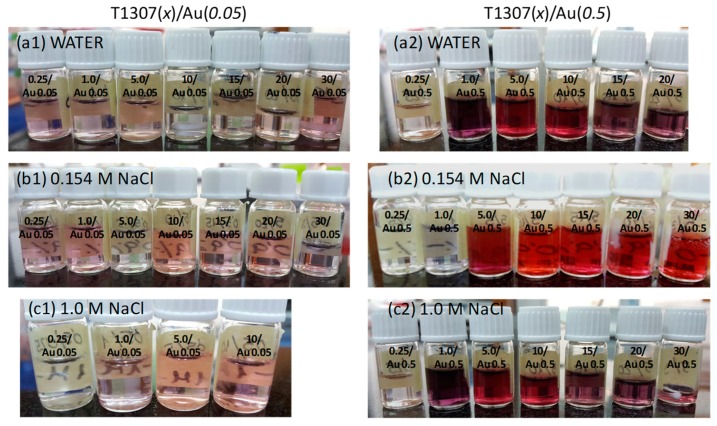
Appearance of T1307/HAuCl_4_ dispersions in water (**a1**,**a2**) or NaCl 0.154 M (**b1**,**b2**) and 1.0 M (**c1**,**c2**) medium after incubation for 4 h at 4 °C. The systems were designed as T1307 (*x*)/Au (*y*) where *x* and *y* represent the concentration (mM) of each component.

Interestingly, mixing T1307 and gold salt caused the initially yellowish dispersions (the color of the starting HAuCl_4_ solution) to begin to exhibit the typical pink-red characteristic color of AuNP formation after few hours of incubation (Figure 1). Nevertheless, relevant differences were observed as a function of T1307, gold, and NaCl concentrations. It should be noted that a further increase in HAuCl_4_ up to 10 mM prevented AuNP formation, probably because the strongly acidic pH of the system (≅2.0) and the lack of sufficient T1307 to trigger the reduction process. Interestingly, when T1307 (1 and 10 mM) containing 10 mM HAuCl_4_ dispersions were diluted with the corresponding T1307 solution, the reduction phenomenon was triggered similarly to the systems initially prepared with less gold salt. This opens the possibility of preparing the T1307/gold salt dispersions in advance, and then the moment at which the reduction (i.e., AuNPs\ formation) should occur may be regulated by adding more copolymer. Relevantly, poloxamine dispersions in water had slightly alkaline pH, and when mixed with the diluted HAuCl_4_ solutions, the pH of the dispersions was in the 6.3 to 7.6 range, which is perfectly suitable for injectable systems. 

**Figure 2:**
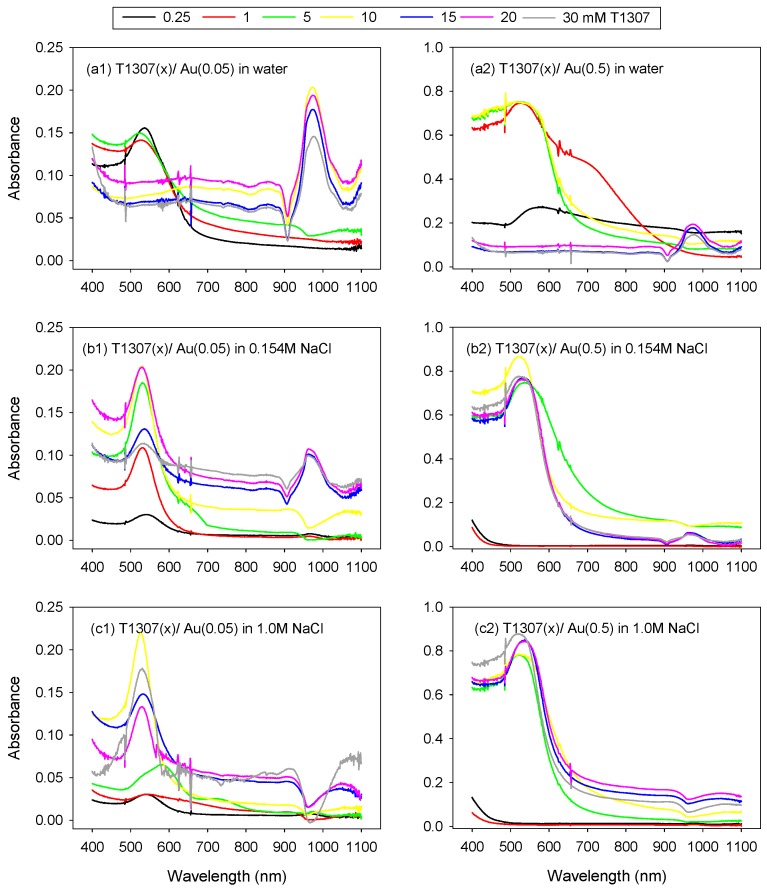
Absorption spectrum of T1307 (0.25, 1, 5, 10, 15, 20, and 30 mM)/Au (0.05 and 0.5 mM) dispersions prepared in (**a**) water, (**b**) 0.154 M NaCl, and (**c**) 1.0 M NaCl aqueous medium. Plots on the left refer to systems prepared with 0.05 mM HAuCl_4_, and plots on the right refer to those prepared with 0.5 mM HAuCl_4_.

Diluted T1307 dispersions (concentration < 10 mM) in water prepared with the lower concentration in gold salt (0.05 mM) showed LPSR peaks centered at ~540 nm, which is typical of the LSPR of spherical AuNPs (Figure 2a1). Differently, after increasing the T1307 concentration, the LSPRs were centered at ~1000 nm, which corresponds to the in-plane resonance of the triangular planar objects [21]; namely, gold nanoplates had been formed (as confirmed below with STEM images). A similar result was observed when the gold salt was added at a ten times higher concentration (Figure 2a2). Spherical gold nanoparticles predominated in dispersions with a T1307 concentration below 10 mM; meanwhile, the LSPR of gold nanoplates was evident for higher T1307 concentrations. The T1307 10 mM concentration seems to be a threshold value; namely, this concentration triggered the rapid formation of nanoplates when 0.05 mM HAuCl_4_ was added, but it was insufficient in the presence of 0.5 mM HAuCl_4_. Indeed, nanosphere LSPR predominated, and only a minor shoulder typical of nanoplates was recorded for T1307 (10 mM)/Au (0.5) in water (Figure 2a2). In a previous work, spherical AuNPs (6–7 nm in diameter) were also reported to be formed using Pluronic L121 (10 mM)/Au (0.25 mM) in water, but nanoplate formation did not occur [19]. In contrast, a low molecular weight poloxamine T904 (MW 6700 Da) triggered the formation of gold triangular and hexagonal nanoplates (174 nm size) at low molar ratio (e.g., T904 (1.5)/Au (0.5)), but after increasing the copolymer concentration, there was a decrease in the number of nanoplates and for T904 (6–140)/Au (0.5), nearly perfect Au nanospheres (up to 40 nm in size) were formed [21]. These shape and size changes recorded for T904 were attributed to the fact that the nanoplate formation benefit from a slow Au (III) to Au (0) reduction rate, which is favored under mild reducing conditions. It has been hypothesized that the selective adsorption of the poloxamine on {111} crystallographic planes of the growing AuNPs due to their lowest energy hinders the growth on these planes and promotes anisotropic growth along the {110} plane, which favors the formation of (111) bounded structures as thin nanoplates [21]. In the case of T1307/Au dispersions, the formation of nanoplates may be favored by the fact that the reaction rate occurred at 4 °C, differently to the incubation at room temperature, or even at a higher temperature, as applied in other protocols [19,21]. Indeed, it was previously observed that heating at up to 70 °C favors rapid and full conversion in spherical nanoparticles [19,21].

Interestingly, the LSPR spectra provided evidence that the addition of NaCl favors nanosphere formation in detriment of nanoplates. In 0.154 M NaCl medium, all T1307 dispersions mixed with the lowest HAuCl_4_ concentration tested led to nanospheres (absorption peak centered in ~540 nm) and the presence of nanoplates was also evident at 15 to 30 mM for T1307 (absorption peak at ~1000 nm) (Figure 2b1). A further increase in HAuCl_4_ concentration (Figure 2b2) revealed that the lowest T1307 concentrations (0.25 and 1 mM) did not trigger significant reduction (as also observed in Figure 1b2); an intermediate T1307 concentration (5–10 mM) favored nanosphere formation; and a further increase in T1307 (15–30 mM) facilitated the coexistence of both nanospheres and nanoplates. Similar results were obtained in 1.0 M NaCl medium, but the LSPR peak of nanoplates attenuated even more (Figure 2c1,c2). It has been previously shown that NaCl causes poloxamers and poloxamines (including T1307) to become more hydrophobic and thus more prone to self-assemble into unimodal micelles [31,32]. Therefore, although Cl^−^ ions have been reported to favor nanoplate formation because of specific adsorption onto crystal facets [33], the decrease in T1307 CMC should result in less unimers being available for reduction and a faster transfer of gold ions into the micelles with the subsequent formation of spherical nanoparticles [21].

### 3.2. AuNP Morphology: STEM and DLS

**Figure 3:**
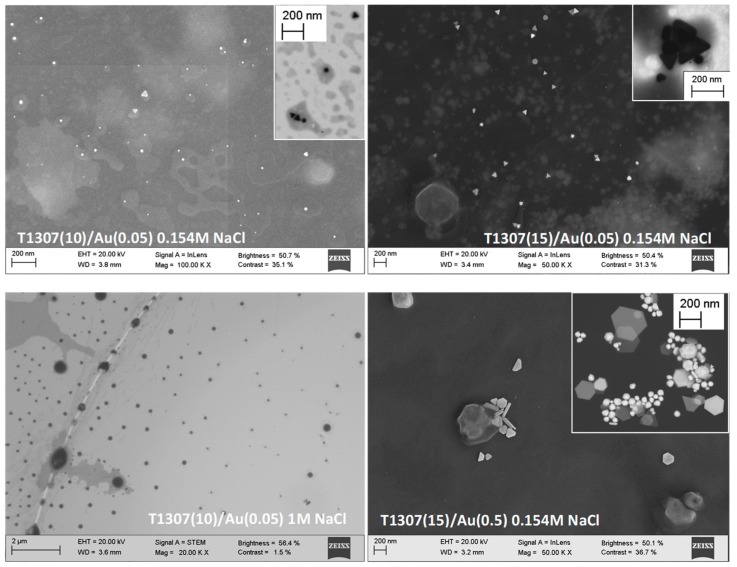
STEM micrographs of T1307 (*x* mM)/Au (*y* mM) dispersions prepared in different media and observed using various detectors (micrographs on the left were recorded using phosphotungstic acid staining, while those on the right were taken without staining).

Scanning transmission electron microscopy (STEM) images allowed visualization of both micelles and AuNPs (Figure 3). Phosphotungstic acid staining provided evidence that the micelles become larger when the NaCl concentration increases (as observed, e.g., for T1307 (10)/Au (0.05) in 1M NaCl) as confirmed by DLS. Both spherical particles and nanoprisms or triangular plates coexisted in the T1307 (10)/Au (0.05) in 0.154 M NaCl, in good agreement with the LSPR spectrum recorded. Interestingly, the AuNPs seem to be encapsulated in the copolymer aggregates and different morphologies of gold particles even coexisted close each other (see insert showing triangles and spheres). Increasing the copolymer and HAuCl_4_ concentration led to gold nanoprisms with sizes predominantly between 140 and 200 nm, also in good agreement with the LSPR spectrum.

The values of hydrodynamic particle size obtained from DLS measurements for T1307/Au dispersions in water and in salt solutions are listed in Table 1. In general terms, the size of the copolymer–AuNP aggregates increased when the copolymer and/or NaCl concentration increased. As observed before, poloxamine aggregation is favored in the presence of NaCl due to a salting-out effect that makes the copolymer become more hydrophobic [31]. This increase in size was particularly evident for T1307 concentrations close to the CMC (0.25–1 mM). The larger sizes recorded for T1307 10 mM systems prepared in 0.154 and 1 M NaCl may be due to the combination of several AuNPs in each copolymer aggregate, as suggested by the STEM images.

### 3.3. Photothermal Measurements

**Figure 4:**
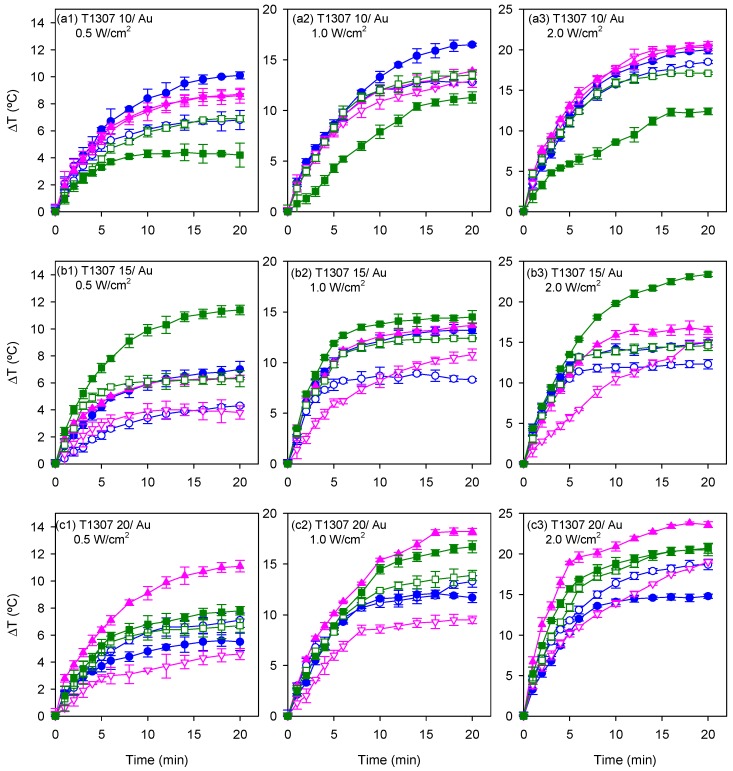
NIR-induced temperature increase (from a basal value of 37 °C) as a function of time for T1307/Au dispersions prepared with (**a**) 10 mM, (**b**) 15 mM, and (**c**) 20 mM T1307, and a final Au concentration of 0.05 (open symbols) or 0.5 (full symbols) mM in water (blue symbols) and in 0.154 M (pink symbols) and 1 M (green symbols) of NaCl aqueous media. The dispersions were exposed to NIR light irradiation using a 980 nm laser at 0.5, 1, and 2 W/cm^2^ for 20 min.

**Figure 5:**
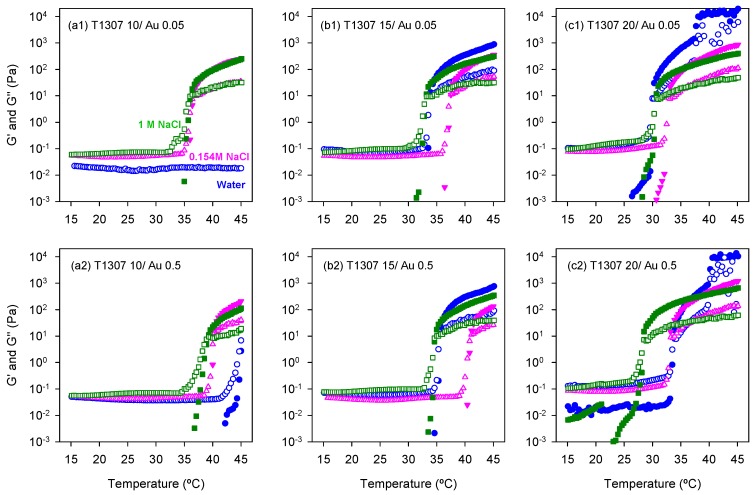
Effect of the temperature on the storage (G’, full symbols) and loss (G’’, open symbols) moduli of (**a**) 10 mM, (**b**) 15 mM, and (**c**) 20 mM T1307 dispersions prepared with 0.05 (1) or 0.5 (2) mM gold salt in water (blue symbols) and in 0.154 M (pink symbols) and 1 M (green symbols) NaCl aqueous solutions.

To evaluate the hyperthermia potential of the AuNPs spontaneously formed in the gelling dispersions, the temperature increase generated during NIR laser irradiation (980 nm) was recorded while maintaining the dispersions in a pre-tempered environment of 37 °C to mimic physiological conditions. The photothermal efficiency was tested using T1307 (10, 15, and 20 mM)/Au (0.05 and 0.5 mM) dispersions in water, and in 0.154 M and 1 M NaCl media, a few hours after the in situ formation of the nanoparticles (i.e., after 4 h storage at 4 °C). These dispersions were irradiated for 20 min at 0.5, 1, and 2 W/cm^2^ power densities. Previously, 20 mM T1307 dispersions (without gold salt) in water and in 0.154 M and 1 M NaCl media were irradiated at 0.5 and 2 W/cm^2^ in order to record the temperature increase of the background, which was 0.4 °C in the first 5 min and 1.1 °C after 30 min of irradiation. Increases of temperature recorded for the systems containing the nanoparticles are shown in Figure 4 and Figure 5 without correction of the background temperature. 

After modifying the concentration of the block copolymer in the dispersion, the photothermal responsiveness varied. T1307 (10 mM)/Au (0.5 mM) dispersions in water achieved the maximum temperature elevation when the laser was applied for 20 min at 0.5, 1, and 2 W/cm^2^ (Figure 4a). An increase in the copolymer concentration gave rise to a modification in the photothermal responsiveness. The increase in temperature after 20 min of irradiation at 2 W/cm^2^ in T1307 (15 mM)/Au (0.5 mM) dispersions in 1 M NaCl medium was 8.4 °C higher (i.e., up to 23.4 °C) than that achieved in the dispersions of the same composition prepared in water (i.e., 15 °C) (Figure 4b). The temperature of T1307 (20 mM)/Au (0.5 mM) dispersions in 0.154 M NaCl increased to 18.9 °C after 5 min of irradiation with the highest power density, and it increased to 23.6 °C after 20 min (Figure 4c). After irradiating for 5 min at the maximum power density, all dispersions achieved a temperature that surpasses the required threshold for the thermal ablation of cancer cells with temperature elevations above 42 °C [34].

Overall, the increases in temperature (with respect a basal value of 37 °C) of 15–20 °C in isotonic medium when irradiated with NIR light of very mild intensity (1 W/cm^2^; typical power densities tested are in 1 to 100 W/cm^2^ range [35,36]) can be considered remarkable and are in good agreement with the formation of non-spherical gold nanostructures. It should be noted that assuming 100% conversion of gold salt into AuNPs, the concentration in gold nanostructures in the T1307/Au 0.05 and 0.5 M dispersions would be 9.85 and 98.5 µg/mL, respectively. These values are in good agreement with the chart residue of the systems recorded by TGA at 450 °C.

It has been previously reported that hybrid polymer AuNPs (15.6 µg/mL) in the form of spheres (12 nm), stars (50 nm), and rods (50 nm) caused increases in temperature of 5, 13, and 18 °C, respectively, upon 1.34 W/cm^2^ light irradiation (continuous wave, λ = 725–2500 nm), which was explained by the progressive shift of the LSPR spectra towards the NIR region [6]. A temperature increase of 10 °C has been reported for pH-induced aggregated gold nanospheres (individual size 33 nm) at 20 µg/mL upon 13.9 W/cm^2^ light irradiation (808 nm) [16]. The increase in thermal efficiency observed when the T1307 concentration increased from 10 to 30 mM for a fixed power density (e.g., 1 W/cm^2^) could be related to the fact that, as discussed below, the T1307 10 mM dispersions did not form gels in this range of temperatures, or the gels formed were weak, whereas T1307 15 mM underwent gelling when heating, and T1307 20 mM were already gels at the basal temperature of the study. In this regard, it has been previously observed that temperature-responsive hybrid elastin-like polypeptide/AuNPs at 120 µg/mL did not show photothermal effects at low temperatures (individual size 30 nm), but caused an increase in temperature of 20–30 °C (irradiation conditions: 808 nm, 1.0 W/cm^2^, 4 min) when they were previously heated (at 30 °C) to form aggregates (940 nm) [17]. Similarly, a clustering effect may be involved in T1037 dispersions under gelling conditions.

### 3.4. Rheological Behavior

Once confirmed that the spontaneously formed AuNPs in the T1307 dispersions are sensitive to NIR, the next step was to investigate whether the dispersions can still undergo sol-to-gel transitions in the presence of the AuNPs and behave as gel depots at body temperature. Rheological characterization of T1307 (10, 15 and 20 mM) dispersions in water and in 0.125 M and 1 M NaCl media with and without gold was carried out in the 15–45 °C range (Figure 5). 

Ten millimole T1307 dispersions prepared in water did not form gels, and the presence of AuNPs did not modify that behavior. This finding is in good agreement with previous reports and means that this hydrophilic copolymer in a good solvent, such as water, requires a higher concentration or further increase in temperature to trigger the micelle self-association [25]. Differently, in 0.154 M and 1 M NaCl media, the sol-to-gel transition of T1307 (10 mM)/Au (0.05 mM) was observed at 36.8 °C and 36.4 °C, respectively, while the transition of T1307 (10 mM)/Au (0.5 mM) occurred at 40.3 and 39.3 °C, respectively (Figure 5a). The shift in the gelling temperature observed in the presence of the highest Au concentration tested (0.5 mM) suggests that some copolymer unimers involved in the stabilization of the AuNPs may be not available to contribute to the micellization and subsequent in situ gelling.

The sol-to-gel transitions of T1307 (15 mM)/Au (0.05 mM) dispersions in water and 0.154 M NaCl and 1 M NaCl media were recorded at 33.9, 37.5, and 33.2 °C, respectively (Figure 5b). These results suggest that the salting-out effect caused by NaCl at a low concentration may cause the PEO corona to be in a less extended conformation, making the contact among micelles more difficult. It should be noted that 0.154 M NaCl caused a minor decrease in the CMC of T1307 compared to that recorded in water [31]. Differently, the CMC was one order of magnitude lower in 1 M NaCl, and thus more unimers are participating in micelle formation and also in the gelling process, which explains the decrease in the gelling temperature. As observed for the less concentrated T1307 dispersions, increasing the Au concentration led to an increase in the gelling temperature. The sol-to-gel transition of T1307 (15 mM)/Au (0.5 mM) dispersions in water and 0.154 M NaCl and 1 M NaCl media occurred at 35.7, 41.1, and 35.0 °C, respectively.

A further increase in T1307 concentration up to 20 mM did not modify the pattern of behavior of the dispersions, but a decrease in the gelling temperature was recorded. T1307 (20 mM)/Au (0.05 mM) dispersions in water and 0.154 M NaCl and 1 M NaCl media underwent the sol-to-gel transition at 30.3, 32.8, and 30.7 °C, respectively, while the gelling temperatures of T1307 (20 mM)/Au (0.5 mM) dispersions in the same media were 33.9, 33.2, and 28.5 °C (Figure 5c). Therefore, in terms of temperature-induced in situ gelling, T1307 (15 mM)/Au (0.05 or 0.5 mM) systems either formulated in water or in 1 M NaCl may be valid as well as any combination of T1307 (20 mM) with Au (0.05 or 0.5 mM). Relevantly, it has been previously shown that similarly highly concentrated T1307 gels (without AuNPs) exhibit a good balance between syringeability, depot consistency, and erosion rate under in vivo conditions [28], which make them suitable for the pursued purpose. 

### 3.5. Performance under Irradiation Cycles at 37 °C

**Figure 6:**
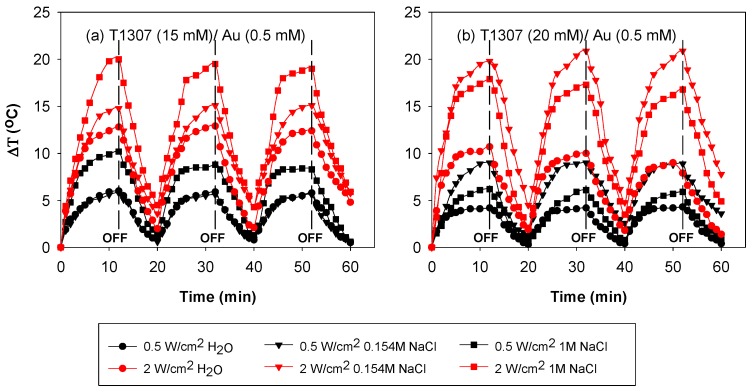
Temperature increase as a function of time under NIR irradiation during three cycles of 12 min irradiation and 8 min of non-irradiation with a 980 nm laser at 0.5 and 2 W/cm^2^ on (**a**) 15 mM T1307 and (**b**) 20 mM T1307 dispersions.

Once the hyperthermia capability of the formulations after a single shot of NIR light was demonstrated, the next step was to elucidate whether the formulations could be useful as depots of gold nanoparticles that provide localized elevations of temperature each time the zone is irradiated. The reproducibility of the photothermal effects has been barely reported in literature [37] and for some materials, irreversible changes and inactivation after the first shot of radiation have been observed [38]. Therefore, for a biomedical application, the reproducibility of the NIR-induced heating should be verified. Consequently, T1307/Au dispersions were subjected to irradiation cycles. The irradiation was maintained until a temperature plateau was achieved. The highest increase in temperature, applying 2 W/cm^2^, was recorded for T1307 (15 mM)/Au (0.5 mM) prepared in 1 M NaCl medium (Figure 6a). In the case of T1307 (20 mM)/Au (0.5 mM) dispersions, the highest increase in temperature was 20 °C when prepared in the isotonic 0.154 M NaCl medium (Figure 4b). The maximum temperature was achieved after 10 min of irradiation. When the laser was switched off, the temperature progressively decreased until near the basal level. Successive laser switch on and off allowed a reproducible response to be recorded. The photothermal effect of all systems tested was maintained after being exposed to three irradiation cycles, which means that the formed AuNPs did not degrade after the first irradiation shot and that they can provide superimposable heating responses (when the laser was on or off). 

## 4. Conclusions

Tetronic 1307 systems have been demonstrated to be able to transform gold salt into AuNPs directly in the final formulation under mild conditions, providing injectable systems with built-in temperature and light responsiveness. Adequate gold salt concentrations are in the 0.05 to 0.5 mM range; higher gold salt proportions may hinder the reduction, probably due to an extremely acid pH and insufficient copolymer. The information gathered points out that (i) the poloxamine concentration plays a key role on AuNPs’ size and shape under isotonic conditions; the typical LSPR of gold nanoplates was recorded for T1307 concentrations equal to or above 10 mM. Interestingly, different morphologies of AuNPs (e.g., triangles, nanoprisms, and spheres) coexist in the same dispersion and seem to be encapsulated in the copolymer aggregates. (ii) The AuNPs cause minor shifts in the temperature-induced gelling transition of T1307 towards slightly higher temperatures, which may be related to the consumption of some copolymer unimers in the stabilization of the AuNPs and, therefore, less unimers are available for the micellization and subsequent in situ gelling. (iii) The spontaneously formed AuNPs exhibit remarkable photothermal responsiveness using a NIR laser of relatively low power, causing increments in temperature which surpass the required threshold for thermal ablation when needed. The gelling of the copolymer dispersion seems to facilitate the efficiency of the responsiveness, probably because of the larger size of the copolymer–AuNP aggregates. Similarly, an increase in NaCl concentration also enhances the photothermal effect. Remarkably, isotonic dispersions of T1307 (15–20 mM) containing AuNPs can undergo the sol-to-gel transition under physiological conditions and may retain the AuNPs for exploitation of the photothermal effects under successive NIR light irradiation cycles. The reproducible photoresponsiveness of the obtained T1307/AuNPs systems may be exploited for ablation of tumor cells or bacteria, but also for other applications such as the photo-controlled release of therapeutic substances.

## Figures and Tables

**Table 1:** Hydrodynamic diameter (nm) recorded at room temperature using DLS for copolymer/gold aggregates formed in T1307/Au dispersions in water or NaCl medium.

[AuCl_4_] mM	[T1307] mM	Ø (nm) in H_2_O	Ø (nm) in 0.154 M NaCl	Ø (nm) in 1 M NaCl
0.05	0.25	2.34 (0.24)	75.8 (21.2)	12.95 (1.63)
	1	35.99 (2.47)	83.6 (21.5)	113.6 (31.2)
	5	68.43 (7.01)	125.9 (23.9)	238.6 (60.5)
	10	116.5 (15.57)	325.8 (65.0)	742.1 (81.9)
0.5	0.25	14.28 (1.01)	102.0 (19.3)	94.5 (20.0)
	1	36.07 (7.93)	194.1 (44.5)	268.5 (58.1)
	5	94.46 (20.02)	284.0 (61.7)	157.3 (62.0)
	10	187.6 (19.89)	466.6 (72.3)	725.8 (98.1)
